# Divergent patterns and drivers of water use efficiency across leaf and ecosystem scales in the Horqin Sandy Land

**DOI:** 10.3389/fpls.2026.1748009

**Published:** 2026-03-12

**Authors:** Xueer Kang, Tingxi Liu, Lina Hao, Limin Duan, Yong Ding

**Affiliations:** 1 Institute of Grassland Research, Chinese Academy of Agricultural Sciences, Hohhot, China; 2 Inner Mongolia Key Laboratory of Grassland Conservation Ecology, Grassland Research Institute, Chinese Academy of Agricultural Sciences, Hohhot, China; 3 Inner Mongolia Agricultural University Water Conservancy and Civil Engineering College, Hohhot, China; 4 Inner Mongolia Key Laboratory of Protection and Utilization of Water Resources, Hohhot, China; 5 Autonomous Region Collaborative Innovation Center for Integrated Management of Water Resources and Water Environment in the Inner Mongolia Reaches of the Yellow River, Hohhot, China

**Keywords:** arid and semi-arid area, eddy covariance system, stable carbon isotope, water use efficiency, water-carbon coupling

## Abstract

**Introduction:**

Water use efficiency (*WUE*) is crucial in the fragile Horqin Sandy Land, yet how global change and ecosystem traits influence its patterns remains unclear.

**Methods:**

This study focused on two ecosystems in the Horqin Sandy Land—semi-mobile dunes with *Artemisia halodendron* (SMAH) and a meadow wetland dominated by *Phragmites australis* (MPA). Using plant carbon isotope ratios and eddy covariance data from the 2022–2023 growing seasons, we revealed the distribution patterns of leaf-scale (*WUE_leaf_*) and ecosystem-scale (*WUE_eco_*) WUE and identified their environmental regulatory mechanisms.

**Results:**

The results showed that the variation trend of *WUE_leaf_* in SMAH closely tracked soil moisture dynamics, whereas *WUE_eco_* lagged behind *WUE_leaf_*, demonstrating that a low T/ET (transpiration to evapotranspiration ratio) plays a key “attenuator” role. In MPA, *WUE_eco_* variation aligned with groundwater level and temperature. Overall, *WUE_leaf_* and *WUE_eco_* responded oppositely to key drivers, but were both abiotic-dominated. *WUE_leaf_* was influenced mainly by soil temperature (with an optimum of 22.8°C) and soil organic carbon, while *WUE_eco_* was driven primarily by net radiation and vegetation cover.

**Discussion:**

*WUE_eco_* variation arose from vegetation–environment interactions rather than vegetation type alone. Thus, *WUE_eco_* cannot be directly extrapolated from *WUE_leaf_*. These results underscore aligning WUE metric selection with research objectives in water–carbon coupling studies, highlighting the role of ecosystem structure and functional traits in regulating water–carbon dynamics.

## 1 Introduction

Drylands rank among the most vulnerable and sensitive terrestrial ecosystems, highly susceptible to environmental changes (Berdugo et al., 2020). Driven by global warming and intensified human activities, the total global extent of drylands is projected to further expand (Alves et al., 2022; Wang et al., 2014), significantly undermining their carbon sink capacity and threatening ecosystem stability (León-González et al., 2023). Future interactions between climate and the carbon cycle may depend more strongly on changes in the water cycle than previously understood (Carvalhais et al., 2014), suggesting that hydrological fluctuations could play an even more critical role in regulating carbon balance and influencing climate change. In recent years, the balance between ecosystem water consumption and productivity has attracted widespread attention (Gentine et al., 2019; Zhang et al., 2023). Water use efficiency (*WUE*), defined as the ratio of carbon fixed by vegetation to total water loss across various temporal and spatial scales (Beer et al., 2009; Morison et al., 2008), reflects water use strategies from leaf to ecosystem levels (Li et al., 2019). *WUE* holds significant value for predicting water and carbon budgets, estimating ecosystem biomass productivity, and assessing changes in ecosystem status, function, and performance under global climate change (Hu et al., 2022; Migliavacca et al., 2021; Poppe Terán et al., 2023). Due to the complex interplay among water, energy, and carbon processes, *WUE* is driven by multiple biotic and abiotic factors and exhibits complex spatiotemporal dynamics across scales (Lavergne et al., 2019; Tan et al., 2015). How *WUE* at different spatiotemporal scales responds to environmental changes remains a central question in current research (Hatfield and Dold, 2019).

Based on observational data, the temporal variation of *WUE* can be quantified using various methods based on a range of factors, including the scale of investigation (from leaf to ecosystem), the frequency of data collection (ranging from half-hourly intervals to interannual trends), the duration of study (from year-to-year variations to multi-year analyses), the nature of the data used (such as plant stable carbon isotope *δ*¹³*C* or eddy covariance flux measurements), and the particular interpretation of *WUE* being applied. Leaf-level *WUE* (*WUE_Leaf_*) is defined as the ratio of photosynthetic rate to transpiration rate (Farquhar et al., 1989b), primarily regulated by stomatal conductance and CO_2_ assimilation rate (Lawson and Vialet-Chabrand, 2019; Yi et al., 2019). It is often estimated via the stable carbon isotope composition (*δ*¹³*C*) of C_3_ plants. Ecosystem-level *WUE* (*WUE_Eco_*) is defined as the ratio of vegetation productivity to evapotranspiration (Yang et al., 2020), typically derived from continuous and synchronous observations of water and carbon fluxes between the canopy and the atmosphere using eddy covariance technology, at daily to seasonal time scalesIt is related not only to plant physiological processes but also to the physical processes of soil evaporation (Jia et al., 2016; Zheng et al., 2019). It is important to note that the two scales differ in terms of water loss. *WUE_Leaf_* typically considers only transpiration through stomata, while *WUE_Eco_* accounts for evapotranspiration, including plant transpiration, soil evaporation, and canopy interception evaporation. Vegetation often occurs in patches, making evaporation a non-negligible component (Xu et al., 2018). In contrast, the canopy-scale water use efficiency (*WUE_Can_*), defined as the ratio of gross primary productivity (*GPP*) to vegetation transpiration (*T*), is primarily linked to plant physiological processes (Scott et al., 2021). It can be expressed as the product of *WUE_Eco_* and *T/ET*, with *T/ET* serving as a key link between *WUE_Can_* and *WUE_Eco_* (Baldocchi et al., 2018). Based on the Penman-Monteith (P-M) model, the Food and Agriculture Organization (FAO) recommends combining crop coefficients with the P-M model to independently estimate evaporation and transpiration; however, its applicability in complex terrain is limited (Ortega-Farias et al., 2010). Compared to the P-M model, the Shuttleworth-Wallace (S-W) model can separately simulate soil evaporation and vegetation transpiration, and it performs well under sparse vegetation conditions due to its accurate representation of evapotranspiration physical mechanisms (Shuttleworth and Wallace, 1985; Hu et al., 2013; Yang et al., 2018). *WUE_Can_* can be regarded as the actual *WUE* of the plant community within an ecosystem, regulated jointly by plant physiology and community structure. As plant communities consist of multiple coexisting species with varying characteristics in carbon uptake and water loss under changing environments, changes in *WUE_Can_* are not a simple sum of changes in *WUE_Leaf_* across all species. Thus, water use efficiency at the leaf, canopy, and ecosystem levels involves distinct carbon and water processes and may exhibit scale-specific responses to climate change.

It is generally accepted that *WUE* values obtained at different scales should be intrinsically related (Medlyn et al., 2017). However, due to fundamental differences in the water and carbon processes involved at leaf and ecosystem scales, physiological responses at the leaf level may be attenuated or amplified when scaled to the ecosystem level through a series of complex biotic and abiotic interactions and feedbacks. These factors encompass alterations in leaf area index and canopy structure, as well as atmospheric boundary layer feedback (Gerten et al., 2008). The interpretation of *WUE_Eco_* is challenged by two main factors: total carbon input incorporates whole-canopy leaf autotrophic respiration (which differs from leaf-scale net assimilation measurements), while the variable proportion of soil evaporation within ET introduces uncertainty in transpiration estimation (Nie et al., 2021). Consequently, scaling the water−carbon processes and their responses to environmental stimuli from the leaf to the ecosystem level remains a critical scientific uncertainty (De Kauwe et al., 2017). Thus, *WUE_Eco_* may not simply represent an upscaled version of *WUE_Leaf_* (Li et al., 2022). For example, based on simulations of climate drivers over the past 50 years in a Mediterranean evergreen forest, Gea-Izquierdo et al. (2015) found that water stress increased *WUE_Leaf_* in dominant plant species but did not lead to a corresponding increase in *WUE_Eco_*. Guerrieri et al. (2016) also observed contrasting trends between *WUE_Leaf_* and *WUE_Eco_* across different vegetation types in North American forests. Medlyn et al. (2017) pointed out an inconsistency in *WUE* trends across global plant functional types when comparing leaf and ecosystem levels. In contrast, Lu and Zhuang (2010) found that moderate water stress could enhance *WUE* in grassland ecosystems, aligning with leaf-scale response patterns. Although existing studies have revealed divergent patterns in *WUE_Leaf_* and *WUE_Eco_* dynamics, a significant knowledge gap remains regarding the mechanisms of their interactions with climate, vegetation, and soil variables. How *WUE_Leaf_* and *WUE_Eco_* synergistically respond to environmental factors in complex ecosystems is still poorly understood.

Dryland ecosystems exhibit high spatial heterogeneity, with significant differences in ecohydrological processes and response patterns across scales (Chen et al., 2022; Hao and Choi, 2023). In the Horqin Sandy Land, sandy and wetland ecosystems experience comparable levels of photosynthetically active radiation and temperature regimes but are distinguished by fundamental differences in vegetation structure and water availability. The dune ecosystem features sparse xerophytic vegetation and a soil moisture regime dominated by deep percolation. In contrast, the meadow ecosystem relies on long-term surface water–groundwater interactions (Wang Y. et al., 2021), which sustain its relatively high productivity (Bao et al., 2025; Delaria et al., 2024). The pronounced difference in water availability leads to distinct growing season lengths: dune plants typically adopt a “fast-growth–dormancy” strategy, rapidly completing their growth during brief moist periods and entering dormancy to endure dry spells. In contrast, although wetland ecosystems benefit from better water supply, they often face combined drought and salinity stress. This multi-abiotic pressure can prompt earlier dormancy, further modulating phenological processes and growth cycles, and resulting in divergent plant functional group composition, surface energy allocation strategies, and biogeochemical cycling, ultimately appearing as cascade ecosystems (Shao et al., 2024). Many existing studies have focused on single ecosystem types, while research from a spatial heterogeneity perspective—particularly along hydrological gradients—remains limited (Reavis et al., 2024; Du et al., 2024; Gea-Izquierdo et al., 2015). The dynamic differences between leaf and ecosystem *WUE* in complex landscape mosaics (e.g., dune-meadow transition zones) are still unclear (Kong et al., 2025; Leifsson et al., 2023). Moreover, the contribution of complex multi-factor effects involving climate characteristics, soil properties, and plant traits to this cross-scale variation in *WUE* remains poorly understood (Maxwell et al., 2018). Elucidating these mechanisms is not only essential for understanding the physiological basis of spatial differentiation in ecosystem functions (Fyllas et al., 2017) but also provides theoretical support for desertification control and ecological restoration in arid and semi-arid regions.

To address this knowledge gap, this study employed stable isotope techniques and eddy covariance technology to reveal the patterns and driving mechanisms of water use efficiency at leaf and ecosystem scales across the gradient ecosystems in the Horqin Sandy Land. The specific objectives are: (1) To investigate the seasonal variations in *WUE* at leaf and ecosystem scales across different ecosystems and their differences during the growing season; (2) To explore the distinct response characteristics of *WUE_Leaf_* and *WUE_Eco_* to environmental factors; (3) To reveal the underlying drivers of multi-scale *WUE* variation in sandy land ecosystems by analyzing its relationships with climatic factors, vegetation dynamics, and soil properties.

## 2 Materials and methods

### 2.1 Study area

**Figure 1:**
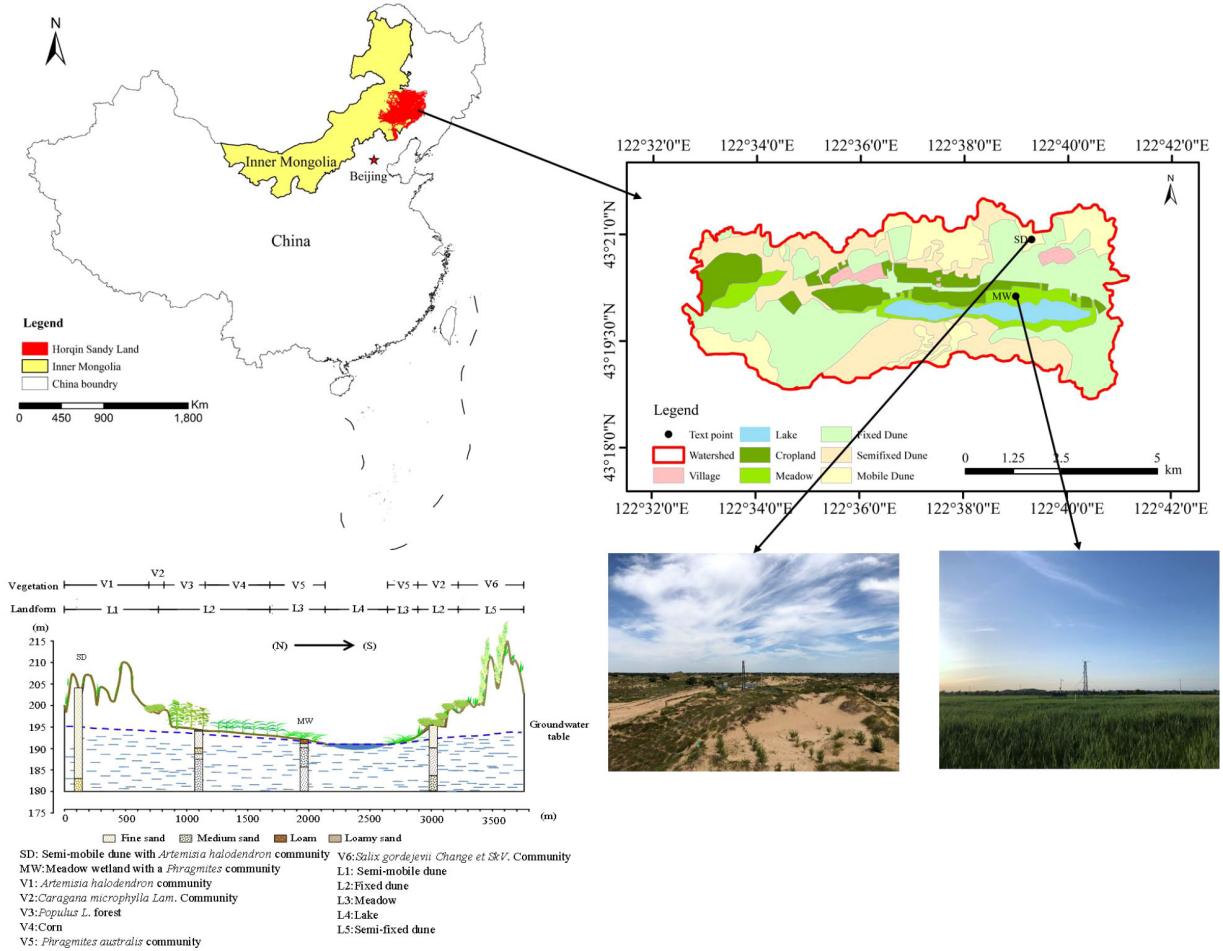
Geographical location of the study area.

The study was conducted at the Agula Eco-Hydrological Experimental Station in the southeastern Horqin Sandy Land (122°33’00”–122°41’’00” E, 43°18’48”–43°21’24”N) (**Figure 1**). Encompassing 55 km², the area features an elevation gradient from 186 m to 232 m. The terrain gradually transitions from south to north, with elevated sand dunes in the southern and northern sections and low-lying farmland and grasslands in the central zone. The region has a semi-arid temperate continental monsoon climate, dominated by southwesterly winds. The mean annual wind speed is 3.6 m/s, mean annual precipitation is 371.1 mm, and mean annual evaporation measured with a Φ20 cm evaporation pan is 1412 mm. The mean annual temperature is 6.6 °C, and the mean annual relative humidity is 55.8%.

### 2.2 Data preparation

#### 2.2.1 Sampling and collection of *δ*^13^*C*

Sample collection was conducted from May to October in 2022 and 2023. Sampling was conducted on typical clear days without precipitation on the sampling day and for the three preceding days. At each landscape unit, four air sampling points were established. Atmospheric CO_2_ samples (200 mL each) were collected, mixed, and sealed in pre-evacuated aluminum bags.

In the semi-mobile dunes comprising *Artemisia halodendron* (SMAH), four fixed standard plants of similar size, representative of the average growth conditions in the area, were selected as monitoring subjects for the entire year. To minimize damage to the plants during sampling, ten living branches approximately 5 mm in diameter were selected from each standard plant during the first sampling round as standard sampling branches. At the beginning of each month, 3–4 mature leaves were collected from each plant.

In the meadow wetland dominated by *Phragmites australis* (MPA), three quadrats (1 m × 1 m) were established. Within each quadrat, 7–8 reed plants were selected as standard sampling subjects. Mature leaves were collected and combined into a single composite sample. The leaves were rinsed with deionized water to remove dust particles, heated at 105 °C for 15 minutes to deactivate enzymes, and then dried in an oven at 65 °C for 48 hours until a constant weight was achieved. Subsequently, the plant material was finely ground using a ball mill and sieved to a particle size of 0.150 mm for *δ*^13^*C* analysis.

Using an elemental analyzer (Vario TOC cube, Elementar, Germany) coupled to a CCIA-38 ER CO_2_ isotope analyzer (Los Gatos Research, USA), we measured the *δ*¹³*C* of atmospheric CO_2_ and plant samples, along with soil organic carbon (*SOC*) and leaf carbon (*LC*) contents. The *δ*¹³*C* abundance, expressed in delta (*δ*) notation (‰), was derived from the following calculation (Equation 1):

(1) $$\delta^{13}C=(\frac{R_{sample}}{R_{standard}}-1)\times 1000‰$$

In this formula, $R_{sample}$ and $R_{standard}$ represent the ^13^*C*/^12^*C* ratios of the sample and the standard, respectively. All *δ*^13^*C* values are reported on the VPDB (Vienna Pee Dee Belemnite) scale.

#### 2.2.2 Monitoring of environmental elements

Both the SMAH and MPA sites were equipped with open-path eddy covariance systems, each comprising a three-dimensional ultrasonic anemometer (CAST3, Campbell Scientific, Inc., USA) and an open-path CO_2_/H_2_O infrared gas analyzer (U-7500A, LI-COR Biosciences, USA). These systems were installed on 10-meter tall flux towers to measure water, heat, and carbon fluxes within the footprint area.

The data logger for the eddy covariance system was a CR3000 (Campbell Scientific, Inc., USA), set to a sampling frequency of 10 Hz. The sensors were mounted on extension arms at heights of 4.9 m (dune site) and 2.7 m (meadow site) above the ground for continuous monitoring of net ecosystem carbon exchange (*NEE*), among other fluxes. Net radiation (*R_n_*) was measured by a four-component net radiometer installed on the tower.

Each site was also equipped with supplementary sensors, including air temperature and relative humidity probes, multi-layer soil temperature and moisture probes, and a rain gauge. All meteorological data—including soil temperature (*T_s_*), soil moisture content (*M_s_*), air temperature (*T_a_*), relative humidity (*RH*), precipitation (*P*), and groundwater depth (*GWD*)—were automatically recorded by a CR1000 data logger (Campbell Scientific, Inc., USA).

Leaf area index (*LAI*) was measured using an LI-2200 plant canopy analyzer (LI-COR Biosciences, USA). Soil *pH* was determined potentiometrically using a pH electrode with a soil-to-water ratio of 1:2.5. Vegetation fractional cover (VFC) was quantified using a standardized digital image analysis protocol. At each sampling point, a downward-facing digital photograph of the ground surface was taken under consistent lighting conditions. All images were processed using ImageJ software (1.8.0; NIH) to objectively separate vegetation from background based on RGB color thresholds. VFC was calculated as the percentage of vegetation pixels within each image. Leaf stomatal conductance (*g_s_*) was measured using an LI-6400 portable photosynthesis system (LI-COR Biosciences, USA).

### 2.3 Sample analyses

The leaf *δ*^13^*C* value can be used to calculate the *WUE_Leaf_* for each sample, based on the well-established relationship between CO_2_ diffusion during photosynthetic isotope discrimination ( $\Delta^{13}C$) and *WUE_Leaf_* (Farquhar and Sharkey, 1982; Farquhar et al., 1989a), as expressed by the following formula (Equation 2):

(2) $$\Delta^{13}C=\frac{\left(\delta^{13}C_{a}-\delta^{12}C_{l}\right)}{\left(1+\delta^{13}C_{l}/1000\right)}$$

In the formula, $\delta^{13}C_{a}$ and $\delta^{12}C_{l}$ represent the carbon isotope composition of atmospheric CO_2_ and the leaf, respectively.

The relationship between *C_i_*/*C_a_* and $\Delta^{13}C$ can also be applied to estimate intrinsic water use efficiency (*iWUE_leaf_*) and *WUE_leaf_* (Barbosa et al., 2010; Ma et al., 2021), which can be expressed as Equation 3:

(3) $$WUE_{\text{leaf}}=\frac{iWUE_{\text{leaf}}}{VPD}=(1-\phi_{c})\times \frac{C_{\text{a}}}{1.6VPD}\times \frac{b-\Delta^{13}C}{b-a+(b-a_{m})\times \frac{g_{s}}{g_{m}}}$$

In the formula, *C_a_* denotes the atmospheric CO_2_ concentration (μmol·mol^-1^), *a* is the diffusion fractionation factor (4.4‰), *b* is the carboxylation fractionation factor (27‰), and the value 1.6 represents the ratio of the diffusion coefficients of CO_2_ and H_2_O in air. The parameter $\phi_{\text{c}}$ at the leaf level is approximated as 0.3 (Evans, 1983). *g_s_* denotes stomatal conductance (mol/m^2^/s), and *g_m_* represents mesophyll conductance (mol/m^2^/s). The ratio *g_s_*/*g_m_* is set to a constant value of 0.79 (Ma et al., 2021), and $a_{\text{m}}$ signifies the isotopic fractionation associated with the dissolution and diffusion of CO_2_ in the mesophyll (1.8‰). *VPD* represents the vapor pressure deficit (mbar) between the intercellular air spaces of the leaf and the surrounding ambient air, calculated as follows (Equation 4):

(4) $$VPD=0.611\times e^{\left[17.502T/(240.97+T_{a})\right]}\times (1-RH)$$

In the formula, *T_a_* represents air temperature (°C), and *RH* represents relative humidity (%).

The TOB1 format data used in this study were converted from raw TOB5 format data. The raw data were preprocessed using EddyPro flux data processing software, which involved the removal of outliers and spurious values, tilt correction, and WPL density correction, resulting in half-hourly flux time series. The REddyProWeb online processing tool (https://www.bgc-jena.mpg.de/bgi/index.php/Services/REddyProcWeb) was then employed to partition *NEE* into ecosystem respiration (*R_eco_*) and gross primary production (*GPP*) following the nighttime partitioning method of Reichstein et al. (2005), which extrapolates daytime *R_eco_* based on the relationship between nighttime *NEE* (i.e., *R_eco_*) and air temperature (Equation 5).

(5) $$NEE=R_{eco}-GPP$$

In the formula, *GPP* represents the CO_2_ absorbed by plant photosynthesis, and *R_eco_* represents the CO_2_ released by both soil and vegetation respiration.

Energy balance closure is a key metric for assessing the performance of an eddy covariance system and the quality of the flux data. It is calculated as follows (Equation 6):

(6) $$\lambda E+H=R_{n}-G-S$$

In the formula, *ΛE* represents the latent heat flux (W/m^2^), *H* is the sensible heat flux (W/m^2^), *R_n_* denotes the net radiation (W/m^2^), *G* is the soil heat flux (W/m^2^), and *S* represents the energy storage term (W/m^2^).

Daily evapotranspiration (*ET*) is calculated using the daily average value of the latent heat flux and converting it using the following formula (Equation 7):

(7) ET=86400*λE/(λρ)

In the formula, the constant 86400 is the conversion factor from seconds to one day, *ρ* is the density of water (1000kg/m^3^), and *Λ* is the latent heat of vaporization (MJ/m^2^). The value of *Λ* was calculated using the daily mean air temperature, *T_a_* (°C), as follows (Equation 8):

(8) $$\lambda =2.501-0.00236*T_{a}$$

*WUE_Eco_* was evaluated as the ratio of *GPP* to *ET* (Moore and Semmens, 2008), expressed as (Equation 9):

(9) $$WUE_{Eco}=\frac{GPP}{ET}$$

In the formula, *GPP* represents gross primary productivity (g Cm^-2^d^-1^), and *ET* represents evapotranspiration (mm).

We calculated the canopy water use efficiency (*WUE_Can_*) for the dune-meadow complex ecosystem using the following formula (Equation 10):

(10) $$WUE_{Can}=\frac{GPP}{T}$$

In this study, the S-W model, integrated with the Ball-Berry stomatal conductance model (Ball et al., 1987) and a light use efficiency model, was employed to estimate evapotranspiration and its components. The detailed algorithm is provided in the **Supplementary Materials**.

### 2.4 Data analyses

Pearson correlation analysis was performed separately between environmental factors and *WUE_Leaf_* and *WUE_Eco_* using the ‘Corrplot’ package in R (version 4.0.2), with the significance level set at P < 0.05. Environmental parameters that showed significant correlations were selected for subsequent analysis, while those directly involved in the calculation of *WUE* itself were excluded.

The Boosted Regression Trees (BRT) model was implemented using the ‘gbm’ package to quantitatively assess the environmental drivers of *WUE* variation across scales. This algorithm enhances model accuracy by constructing multiple regression trees through boosting and random subsampling (Zhou et al., 2020). Partial dependence plots for the top two contributing environmental factors were generated using the partialPlot function from the ‘randomForest’ package, illustrating the marginal effect of individual environmental factors on *WUE* while controlling for other covariates.

Independent samples t-tests (significance threshold P < 0.05) were used to compare differences in *WUE* and their primary environmental drivers between different vegetation types at the leaf scale (herbaceous vs. shrub) and the ecosystem scale (sandy land vs. wetland).

## 3 Results

### 3.1 Hydrothermal conditions of the atmosphere and soil

**Figure 2:**
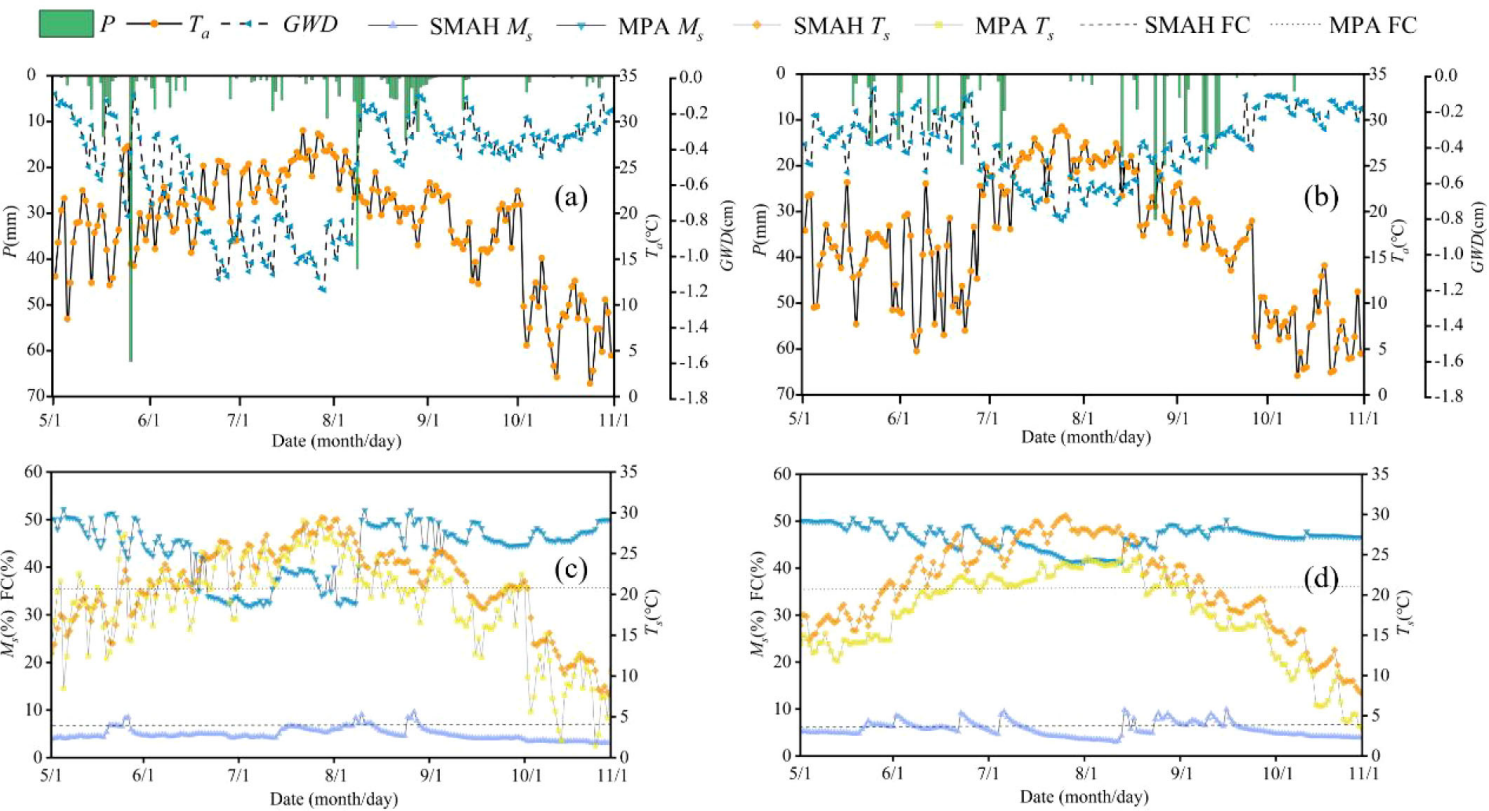
Variations in precipitation (*P*), air temperature (*T_a_*), groundwater depth (*GWD*), soil temperature (*T_s_*), and soil moisture (*M_s_*) during the growing seasons of 2022 and 2023 in the semi-mobile dunes comprising *Artemisia halodendron* (SMAH) and meadow wetland dominated by *Phragmites australis* (MPA). **(a, c)** represent data from 2022; **(b, d)** represent data from 2023.

Total growing season precipitation (*P*) measured 341.7 mm in 2022 and 304.9 mm in 2023 (**Figure 2**). The maximum daily *P* values were recorded on 26 May 2022 (62.4 mm) and 25 August 2023 (31.8 mm). The mean *T_a_* was 17.5 °C in 2022 and 14.9 °C in 2023, with the annual peak temperatures observed on 22 July 2022 (29.04 °C) and 25 July 2023 (29.27 °C).

In the MPA, the *GWD* ranged from 0.087 m to 1.188 m in 2022 and from 0.068 m to 0.805 m in 2023, with annual averages of 0.507 m and 0.386 m, respectively. The *T_s_* dynamics at both the SMAH and MPA sites followed a similar trend to *T_a_*. The mean *T_s_* values in 2022 were 18.63 °C at SMAH and 15.46 °C at MPA, while in 2023 they were 21.29 °C and 17.54 °C, respectively.

The *M_s_* at the SMAH site fluctuated between 3% and 9.9% across both years. In contrast, *M_s_* at the MPA site varied from 31.8% to 52.2%, with values on certain days falling below the field capacity (FC, 36.3%) due to drought and low rainfall conditions.

### 3.2 *WUE_Leaf_* and *WUE_Eco_* variability

**Figure 3:**
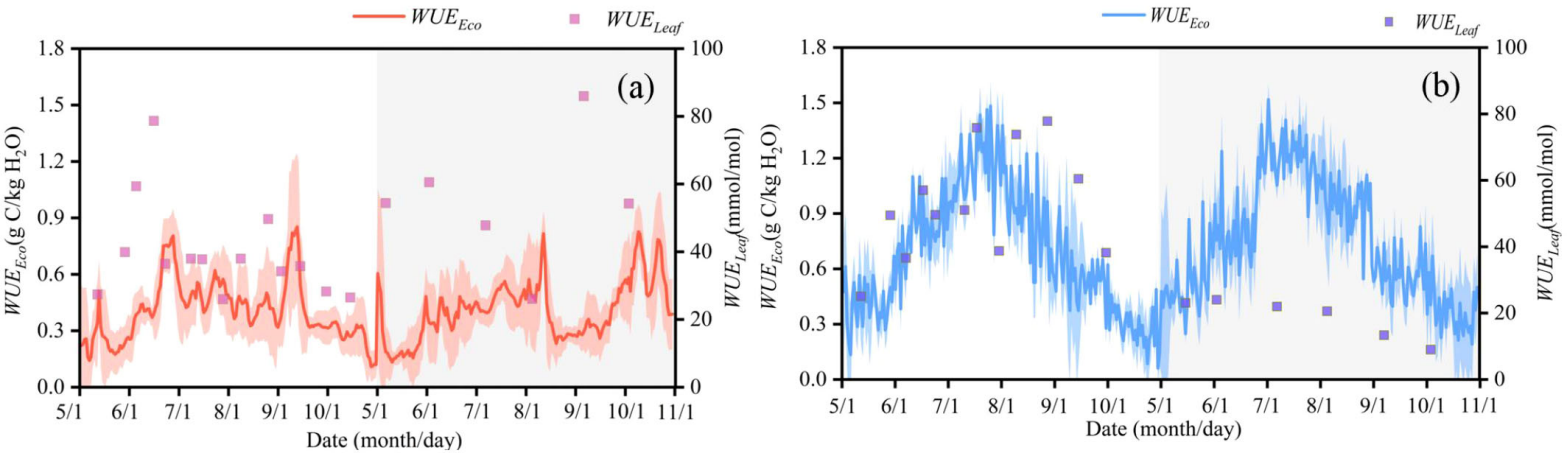
Seasonal changes of leaf water use efficiency (*WUE_Leaf_*) and ecosystem water use efficiency (*WUE_Eco_*) in semi-mobile dunes comprising *Artemisia halodendron* (SMAH) **(a)** and meadow wetland dominated by *Phragmites australis* (MPA) **(b)** in 2022 and 2023. Data from 2022 are shown on a white background, while data from 2023 are indicated by a gray background.

**Figure 4:**
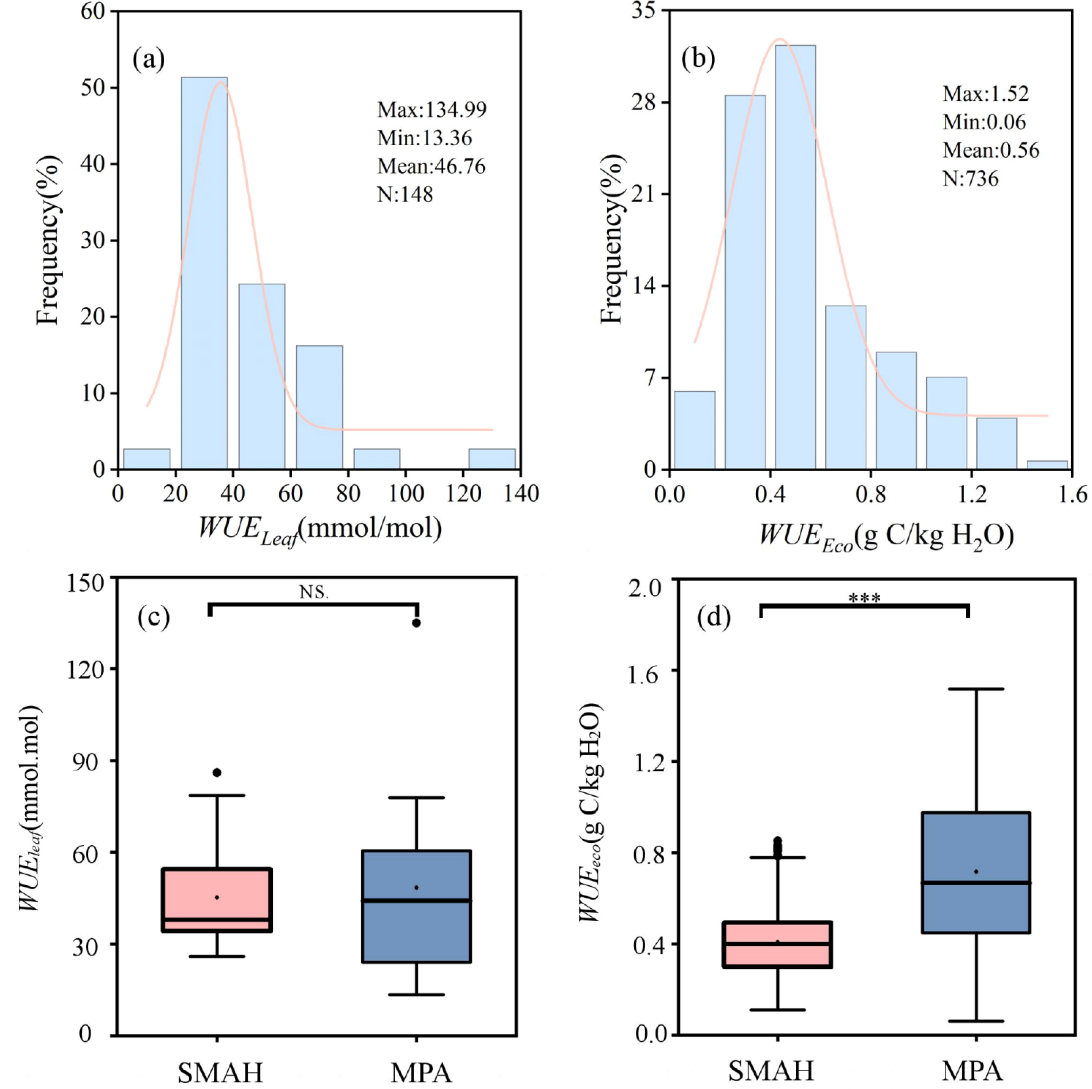
Frequency distribution diagram and box diagram of leaf water use efficiency (WUE_Leaf_) **(a, c)** and ecosystem water use efficiency (WUE_Eco_) **(b, d)** in semi-mobile dunes comprising Artemisia halodendron (SMAH) and meadow wetland dominated by Phragmites australis (MPA). Asterisks indicate statistical significance (***P < 0.001); "NS" denotes not significant.

In 2022 and 2023, in the Horqin Sandy Land, the ranges of *WUE_Leaf_* and *WUE_Eco_* were 13.36–134.99 mmol/mol and 0.06–1.52 g C/kg H_2_O, respectively, with mean values of 46.76 mmol/mol and 0.56 g C/kg H_2_O (**Figure 3**, **Figures 4a, b**). The average values of *WUE_Leaf_* and *WUE_Eco_* in the dune ecosystem are 44.25 mmol/mol and 0.41 g C/kg H_2_O, respectively, whereas in the meadow ecosystem, the average values are 46.35 mmol/mol and 0.72 g C/kg H_2_O. At the leaf scale, the difference in *WUE_Leaf_* between the SMAH and MPA sites was not statistically significant (P > 0.05) (**Figure 4c**). In contrast, at the ecosystem scale, the difference in *WUE_Eco_* between the two sites was highly significant (P < 0.001) (**Figure 4d**), with the mean *WUE_Eco_* value in the MPA being significantly higher than that in the SMAH. The mean *T/ET* values at the SMAH and MPA sites were 0.16 and 0.59, respectively (**Supplementary Figure S1**). In the Horqin Sandy Land, *WUE_Can_* ranged from 0.24 to 4.34 (**Supplementary Figure S2**). The *WUE_Can_* values at the two sampling points are 2.70 and 1.39 g C/kg H_2_O, respectively.The temporal trend of *WUE_Can_* was largely consistent with that of *WUE_Leaf_*, and *WUE_Can_* was consistently greater than *WUE_Eco_*.

### 3.3 Environmental controls on water use efficiency across scales

**Figure 5:**
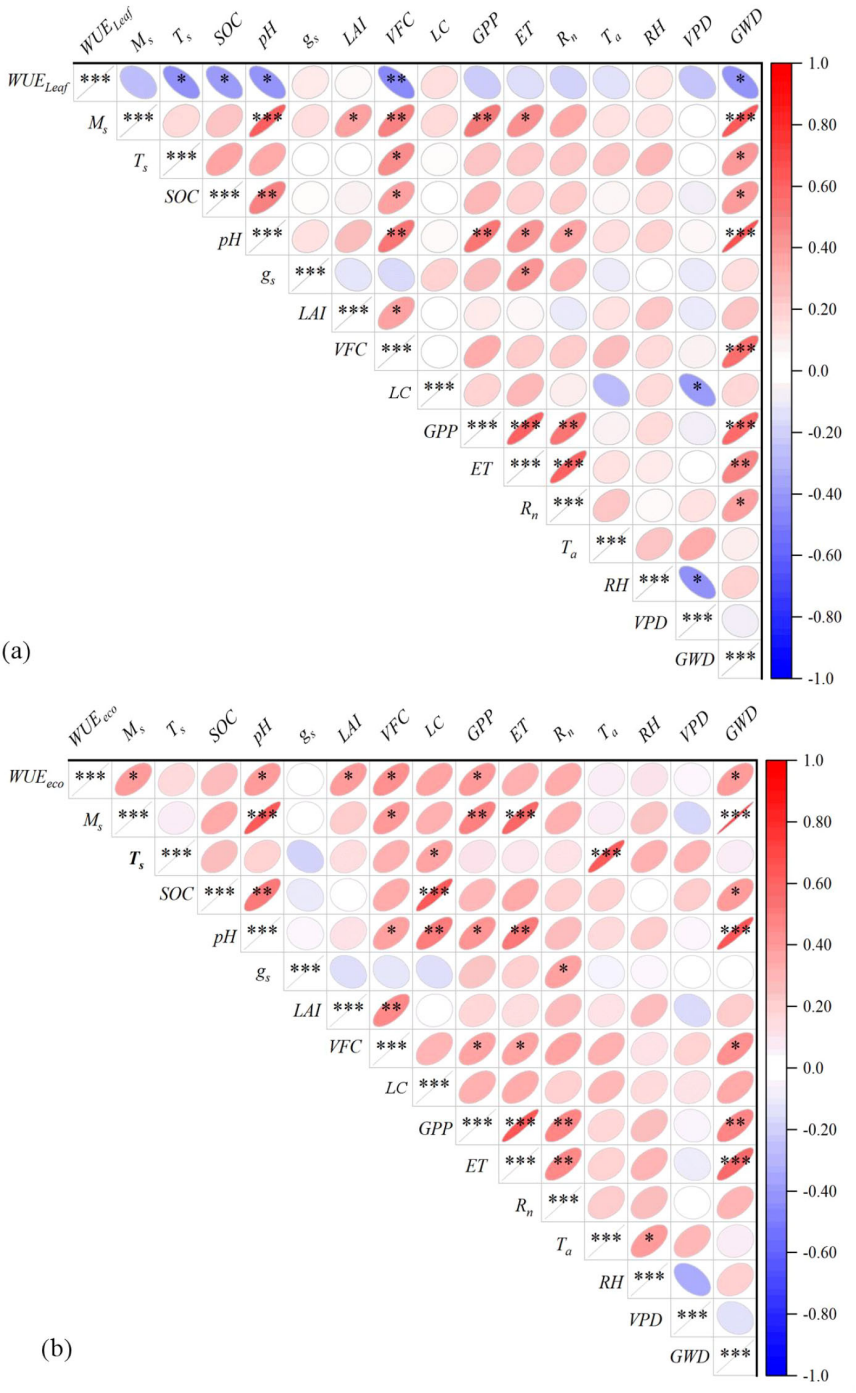
Pearson correlation heat map of leaf water use efficiency (WUELeaf) **(a)** and ecosystem water use efficiency (WUEEco) **(b)** with soil, vegetation and meteorological factors in semi-mobile dunes comprising Artemisia halodendron (SMAH) and meadow wetland dominated by Phragmites australis (MPA). Asterisks indicate statistical significance at probability levels of *P < 0.05, **P < 0.01, and ***P < 0.001.Ms, soil moisture; Ts, soil temperature; SOC, soil organic carbon; gs, leaf stomatal conductance; LAI, leaf area index; VFC, vegetation fractional cover; LC, leaf carbon; GPP, gross primary production; ET, evapotranspiration; Rn, net radiation; Ta, air temperature; RH, relative humidity; VPD, vapor pressure deficit; GWD, groundwater depth. Red and blue colors represent positive and negative correlations, respectively.

At the leaf scale, *WUE_Leaf_* showed significant negative correlations with *T_s_*, *SOC*, *pH*, *VFC*, and *GWD* (**Figure 5a**), but no significant correlations were found with *M_s_*, *g_s_*, *LAI*, *LM*, *GPP*, *ET*, *R_n_*, *T_a_*, *RH*, or *VPD* (vapor pressure deficit) (P > 0.05). In contrast, at the ecosystem scale, *WUE_Eco_* was significantly positively correlated with *M_s_*, *pH*, *LAI*, *VFC*, *GPP*, and *GWD* (**Figure 5b**), while no significant relationships were observed with *T_s_*, *SOC*, *g_s_*, *ET*, *R_n_*, *T_a_*, *RH*, or *VPD* (P > 0.05).

**Figure 6:**
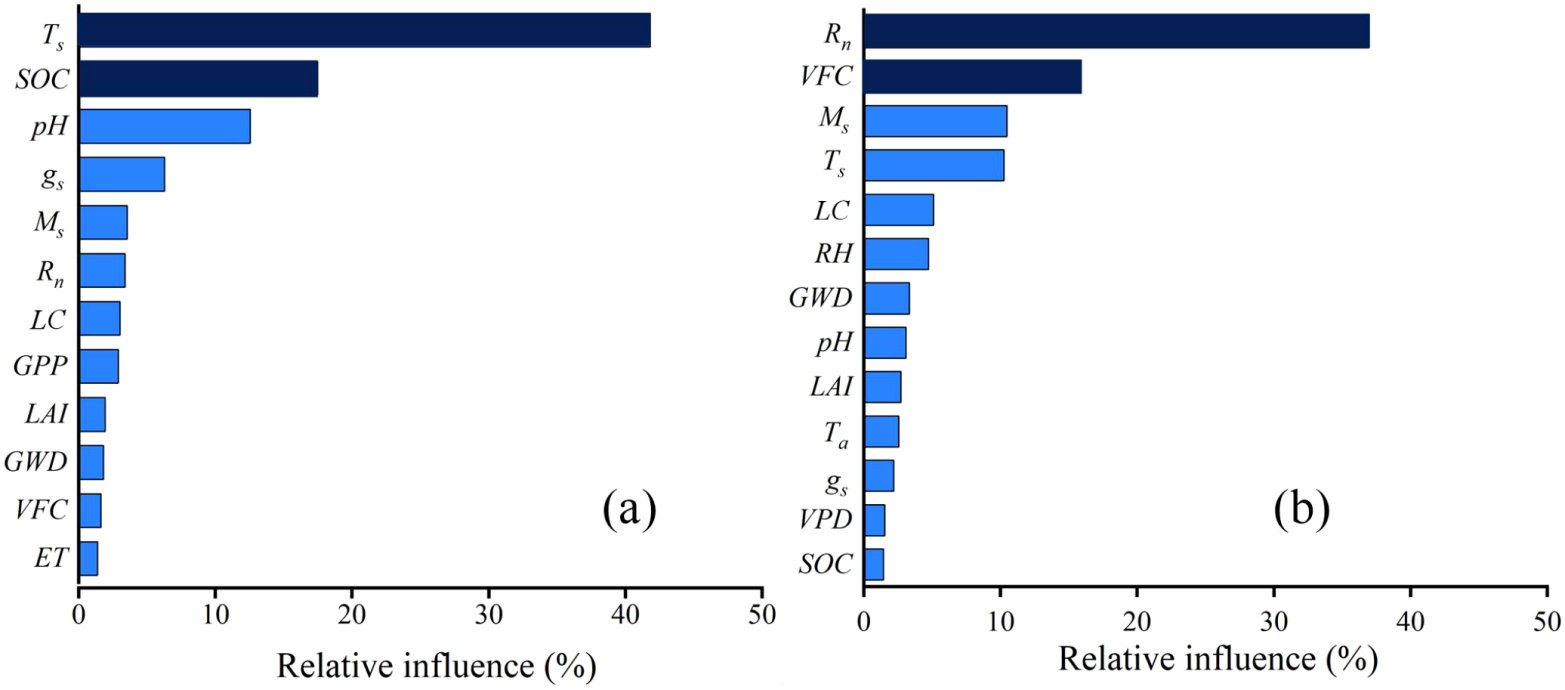
The importance ranking of influencing factors of leaf water use efficiency (*WUE_Leaf_*) **(a)** and ecosystem water use efficiency (*WUE_Eco_*) **(b)** in semi-mobile dunes comprising *Artemisia halodendron* (SMAH) and meadow wetland dominated by *Phragmites australis* (MPA). *M_s_*, soil moisture; *T_s_*, soil temperature; *SOC*, soil organic carbon; *g_s_*, leaf stomatal conductance; *LAI*, leaf area index; *VFC*, vegetation fractional cover; LC, leaf carbon; *GPP*, gross primary production; *ET*, evapotranspiration; *R_n_*, net radiation; *GWD*, groundwater depth. Factors with a ranking less than 2 are shown in dark blue, while those with a ranking greater than 2 are indicated in light blue.

Water use efficiency in the Horqin Sandy Land was closely associated with both biotic and abiotic factors at the leaf and ecosystem scales (**Figure 5**). After excluding variables directly involved in *WUE* calculation, the results indicated that *T_s_* (41.80%) and *M_s_* (17.46%) were the key determinants of *WUE_Leaf_*, with their combined relative influence exceeding 60% (**Figure 6a**). In contrast, *WUE_Eco_* was primarily influenced by *R_n_*(36.95%) and *VFC*(15.84%), which together accounted for over 50% of the total relative influence (**Figure 6b**).

**Figure 7:**
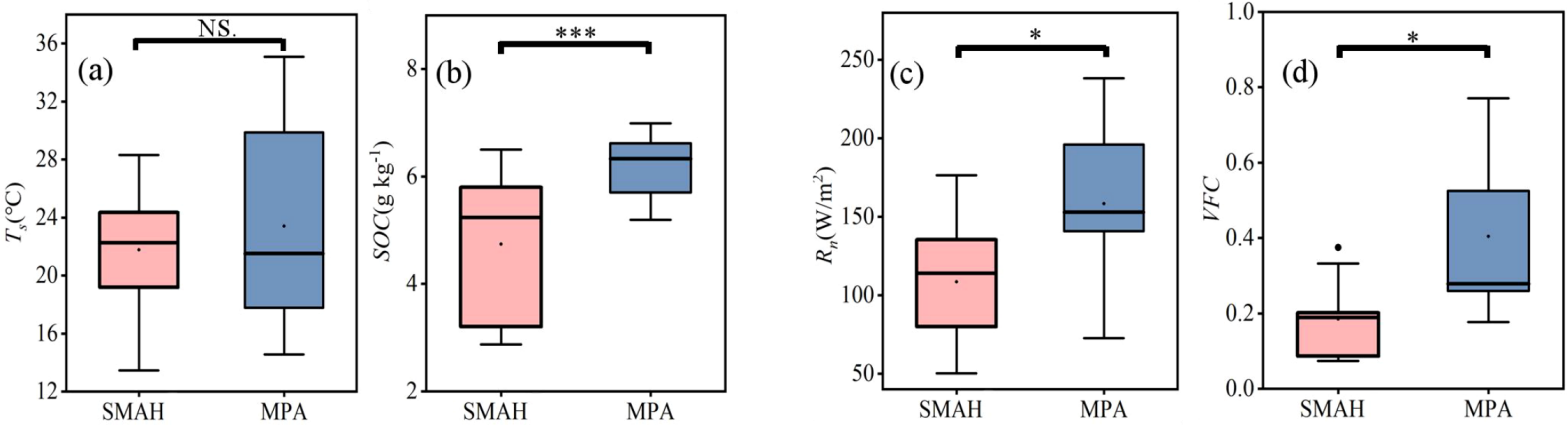
Box diagram of important factors of leaf water use efficiency **(a, b)** and ecosystem water use efficiency **(c, d)** in semi-mobile dunes comprising Artemisia halodendron (SMAH) and meadow wetland dominated by Phragmites australis (MPA). Ts, soil temperature; SOC, soil organic carbon; Rn, net radiation; VFC, vegetation fractional cover; Asterisks indicate statistical significance at probability levels of *P < 0.05, **P < 0.01, and ***P < 0.001. “NS” denotes not significant.

For *WUE_Leaf_*, the difference in *T_s_* between the SMAH and MPA sites was not statistically significant (P > 0.05) (**Figure 7a**), and the *T_s_* ranges overlapped between the two ecosystems. In contrast, the difference in *SOC* between SMAH and MPA was highly significant (P < 0.01) (**Figure 7b**). For *WUE_Eco_*, the mean values of both *R_n_* (158.24 vs. 108.34) and *VFC* (0.40 vs. 0.19) were significantly higher in the MPA than in the SMAH (**Figures 7c, d**).

**Figure 8:**
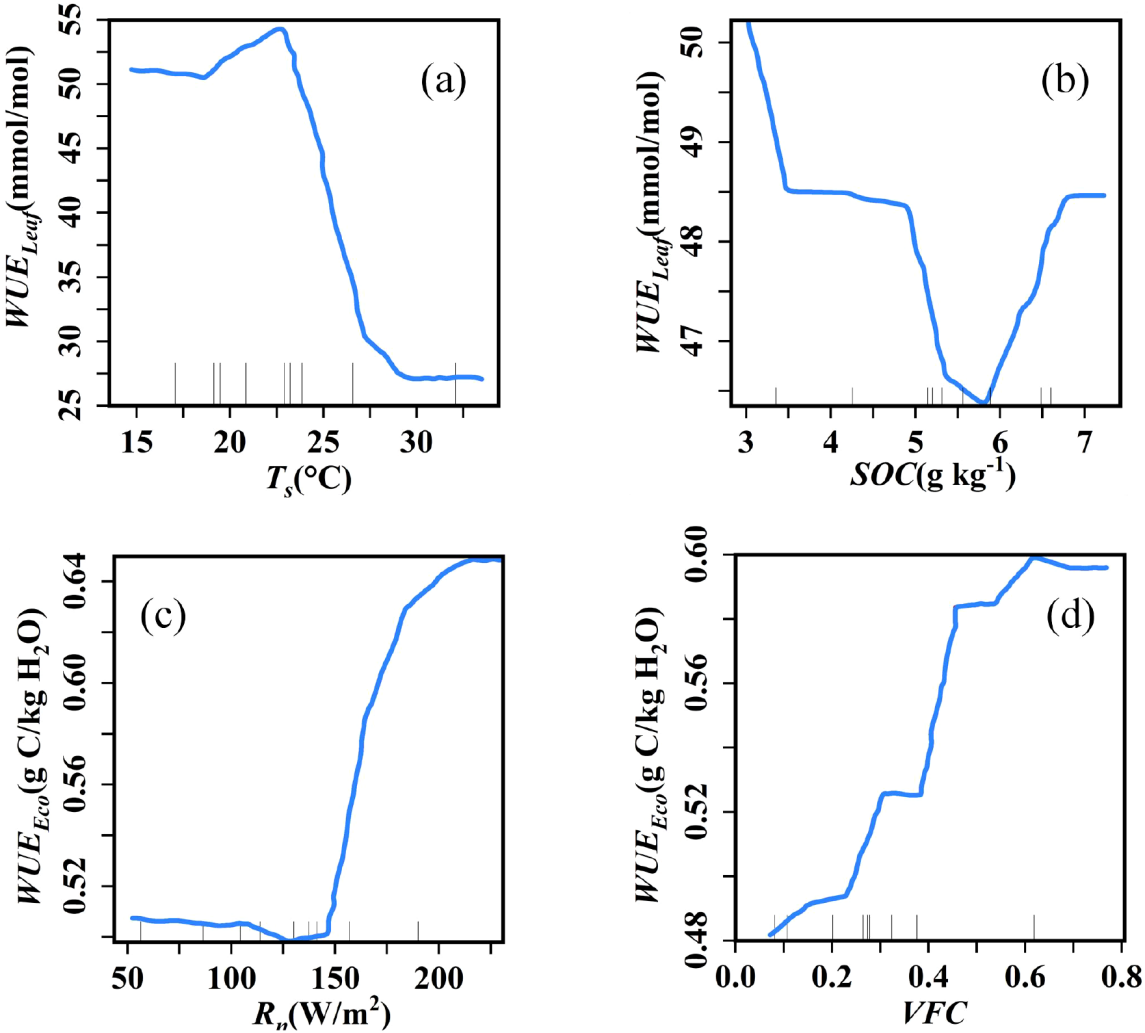
Partial dependence graph of the first two important variables of leaf water use efficiency (*WUE_Leaf_*) **(a, b)** and ecosystem water use efficiency (*WUE_Eco_*) **(c, d)***T_s_*, soil temperature; SOC, soil organic carbon; *R_n_*, net radiation; VFC, vegetation fractional cover.

To further investigate the relationships between key variables and *WUE_Leaf_* and *WUE_Eco_*, partial dependence plots were generated for the top two most important predictors of each, illustrating their marginal effects while controlling for other parameters (**Figure 8**). Specifically, *WUE_Leaf_* showed a positive correlation with *T_s_* within the range of 18.59–22.87 °C, beyond which it decreased significantly as *T_s_* increased from 22.87 °C to 29.04 °C, and then stabilized above 29.04 °C (**Figure 8a**). Meanwhile, *WUE_Leaf_* exhibited a decreasing trend as *SOC* increased from 3.03 to 5.83 g/kg, followed by an increase beyond 5.83 g/kg until it stabilized around 6.75 g/kg (**Figure 8b**).

*WUE_Eco_* decreased slightly as *R_n_* increased from 52.39 to 128.19 W/m², after which it rose sharply with further increases in *R_n_* (**Figure 8c**). In response to *VFC*, *WUE_Eco_* increased rapidly as *VFC* rose from 0.07 to 0.61, then slightly declined and gradually stabilized at higher *VFC* values (**Figure 8d**). *WUE_Eco_* decreased slightly with increasing *R_n_* up to 128.19 W/m², then rose sharply beyond this point (**Figure 8c**). In response to *VFC*, *WUE_Eco_* increased rapidly from 0.07 to 0.61, then slightly declined and stabilized at higher cover values (**Figure 8d**).

## 4 Discussion

### 4.1 Seasonal distribution patterns of *WUE_Leaf_* and *WUE_Eco_*

Under similar climatic conditions, *WUE* varied with vegetation type and ecosystem type (**Figure 3**). In the dune ecosystem, the variation trend of *WUE_Leaf_* largely aligned with soil moisture dynamics, whereas no such pattern was observed in the meadow ecosystem (**Figures 2**, **3**). Deep-rooted dune plants exhibited an opportunistic strategy, capitalizing on ephemeral water to drive a “moisture pulse–sensible heat surge” feedback for rapid photosynthetic activation. Nevertheless, persistent carbon fixation was hampered by the swift loss of soil water (He et al., 2025). Meadow ecosystems, conversely, sustained transpirational cooling by relying on deep soil moisture reserves, ensuring greater stability (Liu et al., 2022). The shallow groundwater table in the meadow wetland ensured sufficient water supply through capillary rise, making FC a decisive factor influencing carbon exchange by mediating moisture inputs from both rainfall and groundwater. Under such conditions, the direction of *WUE* pulses depended on both rainfall amount and pre-rainfall soil moisture content at 0–20 cm depth (Kang et al., 2023). This contrast highlights that overlooking ecosystem specificity may obscure the multidimensional nature of climate response mechanisms in arid regions.

The variation in water use efficiency for both the dune and meadow ecosystems ranged from 0.06 to 1.52 g C/kg H_2_O, which was within the range reported in previous studies (Wang et al., 2023; Hu et al., 2008; Wang H. et al., 2021; Lu and Zhuang, 2010). The *WUE_Eco_* exhibited a pattern of SMAH < MPA, a finding that was consistent with the results of Bao et al. (2025) in the Horqin Sandy Land. This pattern was attributed to the high vegetation cover and sufficient water supply in the meadow ecosystem, which led to a lower proportion of soil evaporation and consequently a higher *WUE_Eco_*. Furthermore, the variation in *WUE_Eco_* in the dune ecosystem exhibited a certain time lag compared to *WUE_Leaf_* and *WUE_Can_* (**Figure 3**). Stomata serve as “valves” regulating gas exchange and water loss in plants, responding rapidly to environmental cues such as light, *VPD*, and soil moisture. Thus, *WUE_Leaf_* and *WUE_Can_* could quickly reflect plant physiological adaptation to immediate conditions. However, *WUE_Eco_* integrated complex ecosystem processes. In the dune ecosystem, a low *T/ET* indicates that a substantial portion of water loss in the sandy land ecosystem originates from soil evaporation (**Supplementary Figure S1**). As a result, the dynamics of *WUE_Eco_* are predominantly controlled by the soil evaporation process. The sparse plant canopy in the dune ecosystem allowed high solar radiation to reach the soil surface, leading to high evaporation rates of surface soil moisture, particularly after rainfall. However, influenced by soil properties and root traits, a water potential gradient between deep roots of sandy shrub vegetation and moist soil layers can drive “hydraulic redistribution, “ where water absorbed by deep roots moves upward and is released into drier surface soil via shallow roots. and the subsequent use of this water in photosynthesis and transpiration—manifested as *GPP* and *ET* fluxes at the ecosystem scale—introduces further delays in transport and physiological processes. Moreover, even when plants closed their stomata in response to water scarcity (reducing transpiration), the evaporation component of evapotranspiration persisted, causing *WUE_Eco_* to respond more sluggishly than *WUE_Leaf_* (Cui et al., 2024). as the surface soil dried rapidly, evaporation decreased sharply and entered a slow phase limited by soil hydraulic conductivity—a process operating on a much longer timescale than stomatal movement. This inevitably results in a lagged response of *WUE_Eco_* compared to surface environmental changes and leaf-level physiological activities directly influenced by precipitation. Consequently, the low *T/ET* in the dune ecosystem acts as a powerful “attenuator, “ severely weakening the transmission of high *WUE_Leaf_ and WUE_Can_* to ecosystem-level performance. Ultimately, this leads to a lagged dynamic in *WUE_ec_*_o_.

In the meadow ecosystem, however, the variation in *WUE_Eco_* closely corresponded with fluctuations in groundwater level and temperature (**Figure 2**, **3**), indicating that when water and energy availability were synchronized, the meadow system achieved higher resource use efficiency and carbon sequestration capacity. In other words, it exhibited greater carbon sink potential by enhancing energy capture and effective water use (Kannenberg et al., 2024). Furthermore, the interannual variation in meadow *WUE_Eco_* was highly synchronized with plant phenology, suggesting that phenological complementarity between C_3_ and C_4_ plants could buffer climate variability through compensatory effects such as differential resource use. Additionally, the high *T/ET* indicates a dominant proportion of vegetation transpiration, resulting in tight coupling between water consumption/utilization and canopy physiological processes (**Supplementary Figure S1**). These characteristics can enhance ecosystem stability. In addition, interannual differences in the relative abundance of plant species in the meadow also led to variations in ecosystem water use efficiency (**Figure 3**). The absence of physiological water deficit stress in wetland vegetation and its resulting high ecosystem *WUE* suggest that these wetlands may face degradation risks under future climate scenarios (Wei et al., 2023).

### 4.2 Controlling factors

In the Horqin Sandy Land, *WUE_Leaf_* and *WUE_Eco_* exhibited contrasting responses to their primary driving factors (**Figure 5**), reflecting fundamentally different water-use strategies operating at distinct organizational scales. In arid ecosystems characterized by sparse vegetation cover (low *VFC*), water loss is dominated by non-productive soil evaporation, resulting in lower *WUE_Eco_*. In contrast, humid areas with denser vegetation experience predominantly effective transpirational water loss (Fatichi and Pappas, 2017; Hao et al., 2023), leading to higher *WUE_Eco_*. Under drought conditions at the leaf level, partial stomatal closure reduces transpiration rates to maintain leaf water content and minimize dehydration risk, while gradually decreasing the photosynthetic rate, thereby increasing *WUE_Leaf_*. This physiological response is consistent with findings that plant species in arid regions exhibit traits associated with higher *WUE_Leaf_* during both photosynthesis and seed germination (Huang et al., 2020). At the ecosystem scale, divergent carbon allocation strategies further explain these contrasting patterns. In arid environments, plants invest substantial carbon resources in survival adaptations—including thick cuticles, sunken stomata, deep root systems, dense palisade tissues, and reproductive structures—while also maintaining essential respiratory costs (Chen et al., 2019). These investments reduce the carbon available for growth-related productivity, consequently lowering *WUE_Eco_*. Conversely, plants in humid regions allocate more fixed carbon to vegetative growth, developing larger leaf areas, wider vessels, and other functional traits that enhance productivity (Huxman et al., 2004). This allocation pattern supports higher plant productivity and consequently higher *WUE_Eco_*. These findings clearly demonstrate that *WUE_Eco_* cannot be simply extrapolated from *WUE_Leaf_* measurements, as they are governed by fundamentally different processes, constraints, and trade-offs across organizational scales.

In terrestrial ecosystems, both biotic and abiotic factors influence carbon and water exchange. These factors not only exhibit significant spatial variability but also considerable temporal and scale variability (Richardson et al., 2007; Li et al., 2022). *WUE_Leaf_* demonstrated greater sensitivity to abiotic drivers (**Figures 5**, **6**), reflecting how water and carbon processes in different species have undergone adaptive evolution to specific environmental conditions. In contrast, *WUE_Eco_* was co-regulated by both biotic and abiotic factors, which is consistent with the global-scale findings of Li et al. (2022). While ecosystem structure and plant traits along climatic gradients can amplify the influence of biotic factors on *WUE_Eco_*, the overall composition of biological communities distributed across environmental gradients may nevertheless result in *WUE_Eco_* exhibiting heightened sensitivity to abiotic drivers (Diefendorf et al., 2010). This phenomenon highlights the capacity of *WUE_Eco_* to aggregate the complex effects of multiple environmental variables on ecosystem processes—from physiological (e.g., photosynthesis, transpiration) to physical (e.g., canopy interception, soil evaporation).

*T_s_* and *SOC* were identified as the primary drivers of *WUE_Leaf_* (**Figure 6**). However, on the Tibetan Plateau in alpine regions, WUELeaf is primarily controlled by air temperature (Wang et al., 2023). This may be because the low temperatures on the Tibetan Plateau limit plant growth, making air temperature the key regulator for initiating photosynthesis and water source thawing. In contrast, the arid conditions and water scarcity in the Horqin Sandy Land mean that plant survival depends on soil moisture, where soil temperature directly governs root water uptake and water availability, thereby dominating *WUE_Leaf_*. The optimal *T_s_* for *WUE_Leaf_* in the study area was 22.8 °C (**Figure 8a**), which falls within the reported optimal Ts range of 15.39–23.89 °C for ecosystem water use efficiency in the Horqin Sandy Land (Zhang et al., 2025). As temperature continued to rise, increased stomatal conductance elevated the intercellular CO_2_ concentration (*P_i_*), while enhanced enzymatic activity improved carboxylation efficiency, thereby reducing *P_i_*. However, the sensitivity of these two processes to *T_s_* differed. In response, plants partially closed stomata and downregulated enzymatic activity to minimize exchange with the external environment—though the extent of these adjustments varied. Therefore, the influence of temperature on *WUE_Leaf_* depended on the relative contributions of stomatal conductance and photosynthetic carboxylases to the ratio of intercellular CO_2_ concentration to atmospheric CO_2_ concentration at a given habitat temperature. This demonstrates the dual role of temperature in regulating the balance between photosynthesis and respiration. Against the backdrop of increasing global extreme drought events, soil temperatures are projected to exceed this optimal threshold more frequently. Such exceeding would reduce *WUE_Leaf_* in sandy land plants and further exacerbate the negative impact of drought on ecosystem carbon sinks. Given the inherent correlation between ecosystem soil moisture and temperature, temperature control and water optimization informed by this relationship can offer valuable guidance for sandy land management. Additionally, the decline in *WUE_Leaf_* below certain hydrological thresholds may reflect a self-regulating, adaptive strategy in desert plants. As a key parameter in models simulating grassland productivity (Niu et al., 2011), integrating species-specific optimal temperature and moisture thresholds for *WUE* could significantly improve the performance of terrestrial ecosystem models under ongoing climate change.

*SOC* emerged as the second most important factor influencing *WUE_Leaf_* (**Figure 6**), with its content following the gradient SMAH < MPA as observed by Kang et al. (2023). Along the transect from SMAH to MPA, vegetation cover, root biomass, and *M_s_* gradually increased, while groundwater depth decreased. Beyond the input of organic carbon from decomposing roots—a product of the MPA’s dense vegetation—the fine-textured, clay- and silt-rich soils play a critical role in long-term sequestration. They enhance the physical and hydrological protection of soil organic carbon, effectively inhibiting microbial decomposition and promoting stabilization (Xu et al., 2016). Therefore, the relationship between *SOC* content and *WUE_Leaf_* was effectively driven by environmental and vegetation differences across the soil organic carbon gradient.

At the ecosystem scale, the top three influencing factors, in descending order of contribution, were *R_n_*, *VFC*, and *M_s_* (**Figure 6**), consistent with the results shown in **Figure 5**. This confirms the critical roles of energy and water availability, as well as canopy development, in governing carbon uptake and water release processes (Hu et al., 2018). In the SMAH site, although *R_n_* increased, the low soil moisture levels limited vegetation water uptake and use, leading to a decrease in *WUE_Eco_* (**Figure 8c**). In this water-limited area, sparse vegetation resulted in lower sensitivity to changes in *R_n_*, thereby weakening the controlling effect of *R_n_* on ecosystem processes, a finding consistent with Kong et al. (2025). Conversely, in the MPA, higher vegetation cover and richer soil organic matter, coupled with lower surface albedo, led to significantly higher surface *R_n_* compared to SMAH (**Figure 7c**). When *R_n_* increased here, vegetation photosynthesis was enhanced. Simultaneously, the higher vegetation cover helped reduce soil water evaporation losses. Consequently, even with increased *R_n_*, soil moisture conditions remained favourable for plant growth and water use, effectively increasing *WUE_Can_* and *WUE_Eco_*. Increased *VFC* at both SMAH and MPA led to greater canopy crowding, which reduced solar radiation reaching the soil surface. This enhanced the overall photosynthetic efficiency of the canopy and, through its direct physiological control linked to canopy conductance and carbon uptake, increased the total productivity per unit ground area and *WUE_Eco_* (Zhao et al., 2021) (**Figure 8d**). Furthermore, increased *VFC* or canopy crowding reduced bare soil water loss, thereby decreasing *E* and increasing *T/ET*. This finding aligns with the study by Bao et al. (2025), which demonstrated that the densification of vegetation in the Horqin Sandy Land affects *WUE_Eco_* by modulating *WUE_Can_* and *T/ET*. These findings provide critical insights for guiding the selection of ecosystem types in arid region restoration, refining the parameterization of water-carbon processes in climate models, and supporting the development of regional carbon neutrality strategies.

### 4.3 Limitations and future directions

This study, through two growing seasons of observations, has preliminarily revealed the divergent patterns and driving mechanisms of multi-scale water use efficiency in the Horqin Sandy Land. However, several limitations remain and should be addressed in future work. Firstly, regarding the data foundation, the conclusions drawn from only two site-years of observation may limit the generalizability of our findings across longer climatic cycles and broader spatial gradients. Secondly, the temporal resolution mismatch between monthly isotope sampling and half-hourly flux data may obscure the upward propagation of leaf physiological responses to instantaneous environmental changes. Furthermore, due to the lack of monitoring of factors such as salinity, it is impossible to comprehensively understand the impact of salinity on the water utilization strategies of wetland plants.

Building upon these limitations, future research can advance in the following concrete directions. First, expanding the observation network by establishing long-term transects across degradation sequences of sandy and meadow ecosystems will help verify the stability of the patterns identified here. Second, conducting synchronous, high−frequency observational experiments during key phenological phases or precipitation events—by coupling leaf gas exchange with ecosystem−scale flux measurements—will enable direct tracing of how physiological signals propagate to ecosystem fluxes. Third, focusing on multi−factor interactions, particularly in ecotones, through designed controlled experiments can clarify the interactive effects of compound stresses, such as water and salinity, on water use efficiency. Collectively, these efforts will contribute to constructing a more robust theory of water−carbon coupling in arid ecosystems and provide more precise scientific and technological support for ecological restoration practices.

## 5 Conclusions

Based on monitoring conducted during the 2022 and 2023 growing seasons in the semi-mobile dune and meadow wetland ecosystems of the Horqin Sandy Land, this study analyzed the seasonal variations in *WUE_Leaf_* and *WUE_Eco_* and their controlling factors, with the aim of elucidating the responses and underlying mechanisms of *WUE* to environmental drivers across scales. The results revealed distinct seasonal patterns in both *WUE_Leaf_* and *WUE_Eco_* between the dune and meadow ecosystems, reflecting divergent physiological mechanisms governing water uptake, transport, transpiration, as well as carbon assimilation, allocation, and respiration during water-carbon flux processes. Climatic, vegetation, and soil conditions not only contributed to these differences but also played a decisive role in modulating the responsiveness of carbon processes to water availability, with the underlying mechanisms varying across vegetation types. We recommend that future conservation and management strategies account for multiple aspects of vegetation and soil conditions across spatial and organizational scales to enhance ecosystem resilience to climate change. Furthermore, management strategies aimed at enhancing the carbon sink function of arid ecosystems should focus concurrently on improving plant physiological water-saving capacity and optimizing ecosystem structure to reduce non-productive water loss (increasing *T/ET*). Further research should emphasize the coupling between vegetation functional types and habitat conditions in shaping terrestrial water-carbon dynamics, with a specific focus on nonlinear feedbacks under multifactorial stress and their integration across scales. Such insights are critical for developing robust predictive models of water and carbon cycling in arid and semi-arid regions.

## Data Availability

The original contributions presented in the study are included in the article/**Supplementary Material**. Further inquiries can be directed to the corresponding authors.
