# Supplementary material for: Divergent patterns and drivers of water use efficiency across leaf and ecosystem scales in the Horqin Sandy Land
Source: Front Plant Sci. 2026 Mar 12;17:1748009. doi: 10.3389/fpls.2026.1748009 (PMC13018148; doi:10.3389/fpls.2026.1748009)
Supplement: Supplementary file 1 [file DataSheet1.docx]

The S-W model is a representative stratified model that separately calculates soil evaporation and canopy transpiration by allocating available energy to the soil surface and canopy surface. The detailed principles of the model are described as follows.

$\lambda ET=\lambda E+\lambda T=C_{s}PM_{s}+C_{c}PM_{c}$ (1)

where $\lambda$ is the latent heat of vaporization (J·kg⁻¹), $C_{s}$ is the soil surface resistance coefficient, $C_{c}$ is the canopy resistance coefficient, $PM_{s}$ is the soil evaporation term, and $PM_{c}$ is the canopy transpiration term.

$PM_{s}=\frac{\Delta A_{t}+\left[ \rho C_{p}D-\Delta r_{a}^{s}\left( A_{t}-A_{s} \right) \right]/(r_{a}^{a}+r_{a}^{s})}{\Delta+\gamma\left[ 1+\frac{r_{s}^{s}}{r_{a}^{a}+r_{a}^{s}} \right]}$ (2)

$PM_{c}=\frac{\Delta A_{t}+\left[ \rho C_{p}D-\Delta r_{a}^{c}A_{s} \right]/(r_{a}^{a}+r_{a}^{c})}{\Delta+\gamma\left[ 1+\frac{r_{s}^{c}}{r_{a}^{a}+r_{a}^{c}} \right]}$ (3)

Where $\Delta$ is the slope of the saturated vapor pressure–temperature curve (kPa·K^-1^), $A_{t}$ and $A_{s}$ represent the available energy at the canopy level and soil surface, respectively; $\gamma$ is the psychrometric constant (0.067 kPa·K^-1^); $\rho$ indicates the air density (1.293 kg·m^-3^); $C_{p}$ denotes the specific heat capacity of air at constant pressure (1012 J·kg⁻¹·K^-1^); $D$ is the vapor pressure deficit (kPa); $r_{a}^{a}$, $r_{a}^{c}$, and $r_{a}^{s}$ correspond to the aerodynamic resistances between canopy height and reference height, within the boundary layer, and between soil surface and canopy height, respectively (all in s·m⁻¹); and $r_{s}^{s}$ and $r_{s}^{c}$ refer to the soil surface resistance and canopy stomatal resistance (s·m⁻¹), respectively.

$A_{t}=R_{n}-G$ (4)

where $R_{n}$ represents the net radiation (W·m^-2^) and $G$ denotes the soil heat flux (W·m^-2^).

$A_{s}=R_{ns}-G$ (5)

$R_{ns}=R_{n}exp(-C_{r}LAI)$ (6)

where $R_{ns}$ denotes the net radiation received at the soil surface and $C_{r}$ represents the extinction coefficient (set to 0.6 in this study). In Equation (1), the two coefficients $C_{c}$ and $C_{s}$ are calculated as follows:

$C_{s}=\frac{R_{c}\left( R_{s}+R_{a} \right)}{R_{s}R_{c}+R_{c}R_{a}+R_{s}R_{a}}=\frac{1}{1+\frac{R_{s}R_{a}}{R_{c}\left( R_{s}+R_{a} \right)}}$ (7)

$C_{c}=\frac{R_{s}\left( R_{c}+R_{a} \right)}{R_{s}R_{c}+R_{c}R_{a}+R_{s}R_{a}}=\frac{1}{1+\frac{R_{c}R_{a}}{R_{s}\left( R_{c}+R_{a} \right)}}$ (8)

where $R_{a}$, $R_{c}$, and $R_{s}$ are calculated as follows:

$R_{a}=\left( \Delta+\gamma\right)r_{a}^{a}$ (9)

$R_{c}=\left( \Delta+\gamma\right)r_{a}^{c}+\gamma r_{s}^{c}$ (10)

$R_{s}=\left( \Delta+\gamma\right)r_{a}^{s}+\gamma r_{s}^{s}$ (11)

The core challenge of the model lies in accurately estimating the canopy stomatal resistance ($r_{s}^{c}$) and soil surface resistance ($r_{s}^{s}$). In this study, the Ball-Berry stomatal conductance model was employed to estimate $r_{s}^{c}$, with the specific formulation given as follows:

$r_{s}^{c}=\frac{1}{g_{0}+a_{1}f\left( \theta\right)P_{n}h_{s}/C_{s}}$ (12)

where $g_{0}$ (μmol·m^-2^·s^-1^) is the minimum stomatal conductance, $a_{1}$ is an empirical parameter, $f\left( \theta\right)$ is a soil moisture function, $P_{n}$ (μmol·m-2·s-1) denotes the net photosynthetic rate,, which in this study is substituted by *GPP*. $h_{s}$ represents the relative humidity at the canopy surface, and $C_{s}$ is the carbon dioxide concentration (ppm).

The functional form of $f\left( \theta\right)$ is given as follows:

$f\left( \theta\right)=\left\{ \begin{aligned} 0, \theta<\theta_{w} \\ \frac{\theta-\theta_{w}}{\theta_{f}-\theta_{w}}, \theta_{w}< \theta< \theta_{f} \\ 1, \theta> \theta_{f} \end{aligned} \right.$ (13)

where $\theta$ denotes the surface soil moisture content, while $\theta_{w}$ and $\theta_{f}$ represent the wilting point and field capacity of the surface soil, respectively.

$r_{s}^{s}$ can be expressed as follows (Lin and Sun, 1983):

$r_{s}^{s}={b_{1}(\frac{\theta_{s}}{\theta})}^{b_{2}}+b_{3}$ (14)

where $b_{1}$, $b_{2}$, and $b_{3}$ are empirical parameters, and $\theta_{s}$ represents the saturated soil moisture content of the surface soil layer

Fig. S1 Seasonal changes of *T/ET* (transpiration divided by evapotranspiration) in semi-mobile dunes comprising *Artemisia halodendron* (SMAH) (a) and meadow wetland dominated by *Phragmites australis* (MPA) (b) in 2022 and 2023. Data from 2022 are shown on a white background, while data from 2023 are indicated by a gray background.

Fig. S2 Seasonal changes of canopy water use efficiency (*WUE_Can_*) in semi-mobile dunes comprising *Artemisia halodendron* (SMAH) (a) and meadow wetland dominated by *Phragmites australis* (MPA) (b) in 2022 and 2023. Data from 2022 are shown on a white background, while data from 2023 are indicated by a gray background.
